# Computer-assisted classification of contrarian claims about climate change

**DOI:** 10.1038/s41598-021-01714-4

**Published:** 2021-11-16

**Authors:** Travis G. Coan, Constantine Boussalis, John Cook, Mirjam O. Nanko

**Affiliations:** 1 Department of Politics and the Exeter Q-Step Centre, University of Exeter, Exeter, UK; 2 Department of Political Science, Trinity College Dublin, Dublin, Ireland; 3 Monash Climate Change Communication Research Hub, Monash University, Melbourne, Australia; 4 Center for Climate Change Communication, George Mason University, Fairfax, USA

**Keywords:** Climate change, Psychology and behaviour, Environmental social sciences

## Abstract

A growing body of scholarship investigates the role of misinformation in shaping the debate on climate change. Our research builds on and extends this literature by (1) developing and validating a comprehensive taxonomy of climate contrarianism, (2) conducting the largest content analysis to date on contrarian claims, (3) developing a computational model to accurately classify specific claims, and (4) drawing on an extensive corpus from conservative think-tank (CTTs) websites and contrarian blogs to construct a detailed history of claims over the past 20 years. Our study finds that the claims utilized by CTTs and contrarian blogs have focused on attacking the integrity of climate science and scientists and, increasingly, has challenged climate policy and renewable energy. We further demonstrate the utility of our approach by exploring the influence of corporate and foundation funding on the production and dissemination of specific contrarian claims.

## Introduction

Organized climate change contrarianism has played a significant role in the spread of misinformation and the delay of meaningful action to mitigate climate change^1^. Research suggests that climate misinformation leads to a number of negative outcomes such as reduced climate literacy^2^, public polarization^3^, canceling out accurate information^4^, reinforcing climate silence^5^, and influencing how scientists engage with the public^6^. While experimental research offers valuable insight into effective interventions for countering misinformation^3,7,8^, researchers increasingly recognize that interdisciplinary approaches are required to develop practical solutions at a scale commensurate with the size of online misinformation efforts^9^. These solutions not only require the ability to categorize relevant contrarian claims at a level of specificity suitable for debunking, but also to achieve these objectives at a scale consistent with the realities of the modern information environment.

An emerging interdisciplinary literature examines the detection and categorization of climate misinformation, with the vast majority relying on manual content analysis. Studies have focused on claims associated with challenges to mainstream positions on climate science (i.e., trend, attribution, and impact contrarianism)^10,11^, doubt about mitigation policies and technologies^12,13^, and outright attacks on the reliability of climate science and scientists^14,15^. Researchers, moreover, have examined the prevalence of contrarian claims in conservative think tank (CTT) communications^14,16^, congressional testimonies^17,18^, fossil fuel industry communications^19^, and legacy and social media^20,21^. Given the significant costs associated with manual approaches for content analysis, several recent studies have explored computational methods for examining climate misinformation, ranging from applications of unsupervised machine learning methods to measure climate themes in conservative think-tank articles^15,22^, to supervised learning of media frames such as economic costs of mitigation policy, free market ideology, and uncertainty^23^.

Our work builds on and extends existing computational approaches by developing a model to detect *specific* contrarian claims, as opposed to broad topics or themes. We develop a comprehensive taxonomy of contrarian claims that is sufficiently detailed to assist in monitoring and counteracting climate contrarianism. We then conduct the largest content analysis of contrarian claims to date on CTTs and blogs—two key cogs in the so-called climate change “denial machine”^24^—and employ these data to train a state-of-the-art deep learning model to classify specific contrarian claims (Methods). Next, we construct a detailed history of climate change contrarianism over the past two decades, based on a corpus of 255,449 documents from 20 prominent CTTs and 33 central contrarian blogs. Lastly, we demonstrate the utility of our computational approach by observing the extent to which funding from “dark money”^25^, the fossil fuel industry, and other conservative donors correlates with the use of particular claims against climate science and policy by CTTs.

## Results

### A taxonomy of climate contrarian claims

**Figure 1:**
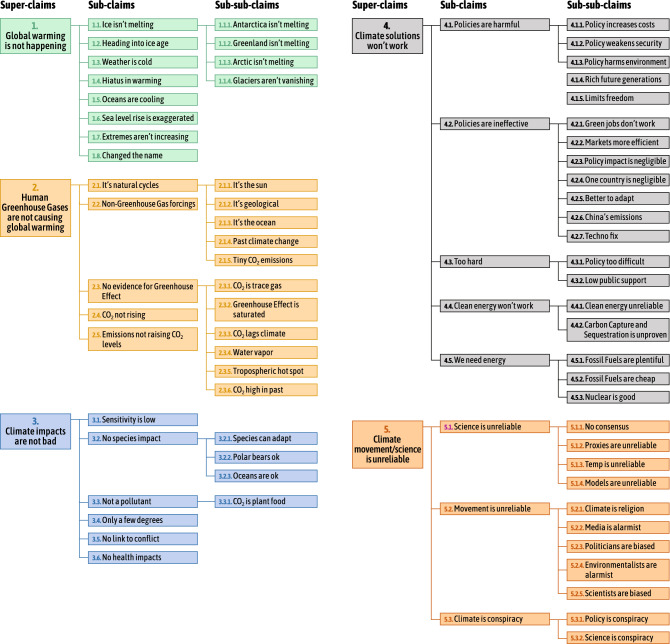
Taxonomy of claims made by contrarians. This figure displays the three layers of claim-making by climate change contrarian actors. The original version of this taxonomy with more detailed claim descriptions can be found in Supplementary Table S2.

Figure 1 displays the taxonomy used to categorize claims about climate science and policy commonly employed by contrarians. To develop this framework, we consulted the extant literature on climate misinformation to identify relevant claims, while further extending and refining this initial set by reading thousands of randomly selected English language paragraphs from prominent CTTs and contrarian blogs (see Supplementary Tables S4 and S5). This process yielded five major categories: (1) it’s not happening, (2) it’s not us, (2) it’s not bad, (4) solutions won’t work, and (5) climate science/scientists are unreliable. We describe these categories as the five key climate disbeliefs, mirroring the five key climate beliefs identified in survey research^26^. Nested within these top-level categories were two sub-levels (27 sub-claims, 49 sub-sub-claims), allowing a detailed delineation of different specific arguments (see Supplementary Methods for additional information on how we developed the taxonomy). This work is, to our knowledge, the first framework incorporating climate science misinformation, arguments against climate solutions, and attacks undermining climate science and scientists in a single, comprehensive taxonomy.

Yet developing a comprehensive taxonomy presents a number of conceptual challenges. Distinguishing between claims that are best described as skeptical (i.e., expressing a reasonable level of doubt based on available evidence), contrarian (i.e., contrary to mainstream views), or outright climate misinformation is a demanding task^27^. The claims presented in Fig. 1 generally fall into three groups. First, many of the claims—and the majority of claims in categories 1–3—have been shown to contain reasoning fallacies^28^ and thus may be confidently labeled as misinformation. Second, a number of claims are factual statements and are only contrarian when used as a rhetorical device to express doubts on the scientific basis for climate change and the need to take policy action. For instance, the “weather is cold somewhere on a certain day” may be factually correct, while “the weather is cold today, therefore global warming is not happening” is not. And while climate models are uncertain, this does not imply that all climate science is unreliable. Lastly, our taxonomy includes several claims that well-known contrarians tend to make, yet are not necessarily contrary to mainstream views. For instance, both contrarian and mainstream advocates have expressed concerns that “CCS is unproven” (4.4.2), and a number of scholars argue that nuclear energy (4.5.3) can make significant contributions to climate mitigation efforts^29^. It is important to note, however, that claims which fall into this third group constitute only a small share of the total claims in the taxonomy; the overwhelming majority of claims directly challenge mainstream views on climate science and policy.

### Climate change contrarianism over the past two decades

We developed custom software to harvest all English language textual content from 33 prominent climate contrarian blogs and the climate-related content of 20 conservative think-tanks over the period from 1998 to 2020. Supplementary Tables S4 and S5 provide a full list of the blogs and CTTs included in this study, as well as the number of documents provided by each source. In total, we collected 255,449 climate change relevant documents—which contain over 174 million words (tokens)—from these 53 sources over the studied time period. Almost all of the CTTs (95%) and the majority of blogs (64%) were from the United States. The only non-US CTT was Canadian while there were a number of non-US blogs (Australia, 12%; Iceland, 6%; New Zealand, 6%; Canada, 3%; Czech Republic, 3%, Germany, 3%; and UK, 3%). Supplementary Figure S1 illustrates the total document frequencies over time, offering the monthly counts of documents for blogs and CTTs.

The 20 most prominent CTTs were identified in previous literature on organized climate contrarianism^14,15^. The selection criteria of the 33 contrarian blogs were based on (1) the list of central contrarian actors presented by Sharman^30^ and (2) the Alexa Rank for each blog. Note that the Alexa Rank score is calculated based on the number of daily visitors and pageviews over a rolling 3 month period. The score provides a rough estimate of the popularity of a particular website. While our list of blogs $(n = 33)$ does not capture the entire contrarian blogosphere, it does cover a large proportion of the movement’s most prominent actors, including 139,912 blog posts over the period 1998 to 2020.

With these data in hand, we adopted a supervised learning approach to classify relevant claims by (1) employing a team of climate-literate coders to categorize a sample of 87, 178 paragraphs along the three levels specified in our taxonomy (Methods and Supplementary Methods) and (2) training a model to accurately classify around 4.6M paragraphs from our corpus of contrarian blogs and CTTs.

**Figure 2:**
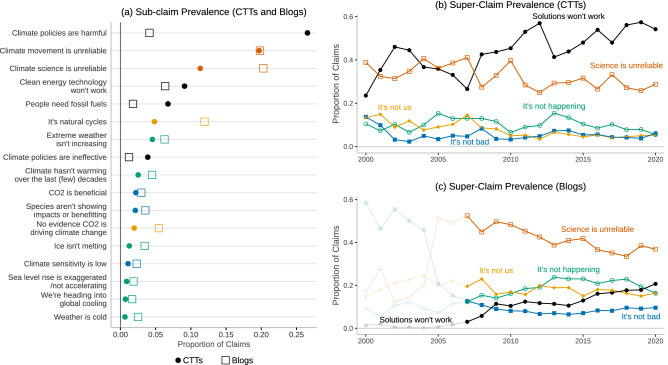
Prevalence of super- and sub-claims by CTTs and contrarian blogs. (**a**) illustrates the share of claim-making paragraphs related to the sub-claims of our taxonomy by CTTs (circle) and blogs (hollow square). (**b**) and (**c**) Display the share of 515,005 claim-making paragraphs devoted to the following super-claim categories: 1. Global warming is not happening (green hollow circle), 2. Humans are not causing global warming (yellow diamond), 3. Climate impacts are not bad (blue filled square), 4. Climate solutions won’t work (black circle), and 5. Climate movement/science is unreliable (orange hollow square). Note that estimates prior to 2007 in (**c**) are derived from a relatively small number of blogs.

Figure 2 provides the prevalence of the five key climate disbeliefs for CTTs (Fig 2b) and blogs (Fig 2c) over time, while also providing the distribution of claim prevalence across relevant sub-claims (Fig 2a). The figure offers insights into the key similarities and differences in claims across contrarian blogs and CTTs, as well as the evolution of claims over time. In general, CTTs focus predominantly on the shortcomings of climate solutions (category 4) and attacks on climate science and scientists (category 5). While the initial years of the series were marked with approximately equal levels of emphasis on these two categories, category 4 gained prominence following 2008. This shift in the focus of the (mainly US-based) CTTs coincides with the transition of power from Republican to Democratic hands and the corresponding threat of climate legislation: in 2007, for the first time since 1993, the Democrats obtained a majority in both congressional chambers and in 2008 Senator Obama, consistently leading the presidential election opinion polls, promised comprehensive climate legislation in his presidential campaign. However, category 4 claims have dominated the CTT discourse for the remainder of the sample period, indicating a more permanent shift towards attacks on climate solutions. Blogs, on the other hand, have consistently devoted the largest share of their claims to attacking climate science and scientists. Yet, even for blogs, discussion of climate policy has risen over the last decade while challenges to the reliability of climate science and the climate movement have been on a downward trend, indicating that future contrarian claims are likely to increasingly focus on climate solutions.

For both CTTs and blogs, claims which outright deny the existence and severity of anthropogenic climate change (categories 1–3) have been stable or have declined in relative terms in recent years. Claims for categories 1–3 are much more likely to be present in blogs than in CTT materials, although the pre-2010 period exhibited non-trivial levels of these claims even among CTTs. These results suggest that the blogs seem to be acting as the pseudo-scientific arm of the climate change counter-movement, with authors from this corpus being more likely to offer alternative explanations for scientific observations and predictions found within the climate science literature. This result is consistent with social network analysis finding the most central networked contrarian blogs are focused on science rather than policy^30^.

A significant advantage of our model is that it can detect claims at a more granular level, which allows us to determine which lower-level claims are driving the macro disbelief trends described above. Figure 2a visualizes the prevalence of selected sub-claims over the entire time period in CTTs (circles) and blogs (boxes), with the list sorted by CTT sub-claim prevalence. Here, we see how the driver of the category 4 arguments made by CTTs has been the claim that mitigation and adaptation measures will be harmful to the economy, environment, and society more generally. Category 5 claims were also prominent in both corpora; however attacks on the science and the climate movement were roughly equally frequent among the blogs, whereas CTTs were more likely to focus on attacking the movement by accusing climate scientists and activists of being alarmist and biased. Note that due to the thematic overlap between sub-claims 5.2 (Movement is unreliable) and 5.3 (Climate is a conspiracy), we collapsed these claims into a single measure both when training our model and presenting results. Further, our results show how the most common sub-claim for both CTTs and blogs not covered by categories 4 or 5 is that observed climate change is simply due to natural cycles.

### A closer look at conservative think tank climate messaging

**Figure 3:**
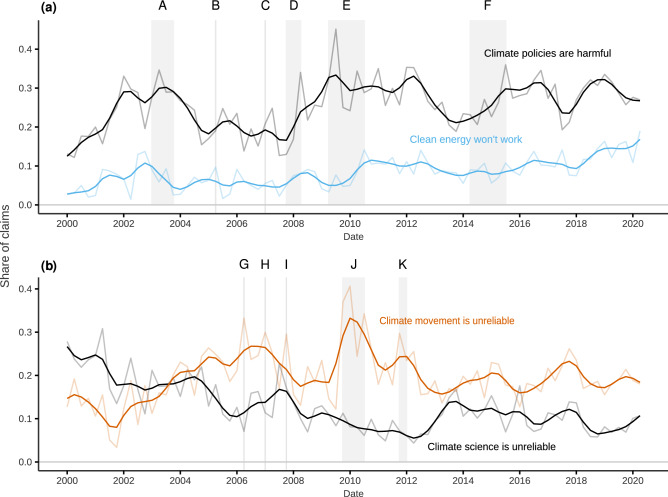
Prevalence of selected contrarian sub-claims in CTT communication. This figure illustrates the temporal variation (quarterly) in the proportion of sub-claims found in CTT documents related to (**a**) “Climate policies are harmful”, “Clean energy won’t work”, and (**b**) “Climate movement is unreliable”, “Climate science is unreliable”. Highlighted periods in the time series include: (A) 2003 Climate Stewardship Act; (B, C) 2005 and 2007 Climate Stewardship and Innovation Acts; (D) Climate Security Act of 2007; (E) American Clean Energy and Security Act; (F) Clean Power Plan; (G-I) *An Inconvenient Truth* and Al Gore Nobel/IPCC Prize; (J) “Climategate”; and (K) Peter Gleick/Heartland Institute affair. Note that darker lines represent cubic splines used to aid interpretation.

Next, given the considerable attention paid to CTT discourse in the literature on organized climate contrarianism^14,15,22,24,31,32^, we offer a more detailed examination of the specific claims of these organizations over two decades. Figure 3a examines the dynamics of two prominent policy-related sub-claims—“Climate policies are harmful” and “Clean energy won’t work”—while also overlaying major US climate policy events, from the 2003 Climate Stewardship Act to the Obama administration’s Clean Power Plan. The highlighted sections of Fig. 3a indicate the relevant beginning and ending dates for these efforts, with the most common being the introduction of and voting on a Congressional bill. The figure demonstrates that claims on the harmful effects of climate policy, particularly for the economy, closely align with changes in the US policy environment: CTTs tend to first ramp up discussion following the announcement of a bill, and then again prior to a bill reaching the floor for a vote. Particularly salient is the spike in policy claims in late 2009, which not only coincided with intense debate on the American Clean Energy and Security Act (ACES), but also with the COP15 climate summit in Copenhagen. This summit was billed as an especially consequential meeting for progressing mitigation policies. Claims that challenge the efficacy of clean energy, however, appear less sensitive to policy events and yet have increased considerably over time, with the second quarter of 2020 representing the highest share of these claims to date. Notably, this trend runs counter to the plummeting cost of renewable energy production^33^.

Figure 3b similarly displays the dynamics of the two leading science-related claims: “Climate movement is unreliable” and “Climate science is unreliable”. Consistent with qualitative accounts of the “denial machine”^34^, in the early 2000s CTTs continued to “manufacture uncertainty”^24^ surrounding scientific evidence on anthropogenic global warming, including questioning the validity of climate models and data. However, while challenging scientific models, data, and the consensus remains a common rhetorical strategy even today (roughly 10% of claims), our data highlight a clear transition in 2005 towards accusations of alarmism, bias, hypocrisy, conspiracy, and corruption against climate scientists, advocates, the media, and politicians. A steady upward trend in these types of claims is seen throughout the George W. Bush administration, with an initial peak between 2006 and 2007. This period was a watershed moment for climate advocacy with the release of *An Inconvenient Truth* and its subsequent Academy Award, the awarding of the Nobel Peace Prize to Al Gore and the IPCC, as well as the publication of a landmark report by the Union of Concerned Scientists criticizing the climate contrarian countermovement. However, the series does not peak again until the so-called “Climategate” controversy in late 2009 (timed to occur a short time before the COP15 summit presumably to undermine climate negotiations) and early 2010^35^, with a smaller subsequent spike in late 2011 following strong reactions to the release of Heartland Institute internal documents by the climate scientist Peter Gleick^36^. While this series has not returned to Climategate-era levels, the “Climate movement is unreliable” category remains a central motif of CTT climate-related messaging.

**Figure 4:**
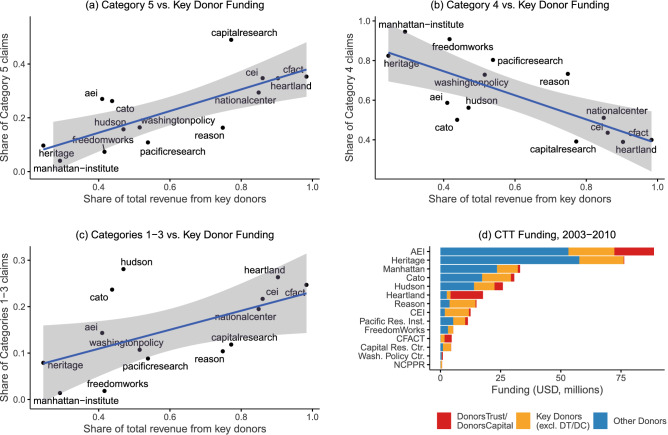
CTT super-claim prevalence and funding from key donors. This figure includes scatterplots and linear regression results (see Supplementary Table S6 for the full results) showing the relationship between the share of CTT funding from “key” conservative donors and the prevalence of claims from the following categories: (**a**) “Climate movement/science is unreliable” [Category 5] ($\beta =0.403$, $p < 0.05$, $R^{2}=0.56$), (**b**) “Climate solutions won’t work” [Category 4] ($\beta =-0.608$, $p < 0.05$, $R^{2}=0.56$), and (**c**) “Global warming is not happening”, “Human GHGs are not causing global warming” & “Climate impacts are not bad” [Categories 1–3] ($\beta =0.205$, $p < 0.05$, $R^{2}=0.25$). Total funding in millions of US dollars over the period 2003-2010 is displayed in (**d**) along with the share of funding from DonorsTrust/DonorsCapital (red), key donors other than DonorsTrust/DonorsCapital (yellow), and other donors (blue).

Moving beyond a description of the dynamics of contrarian claims, our data also offers the ability to explore salient relationships between contrarian content and other features of the climate change countermovement. One important area of climate research on organized climate contrarianism is the influence of conservative interest group funding on the production and dissemination of climate change misinformation by actors within the counter-movement^24,25,37^. While existing work has demonstrated how corporate funding is correlated with particular climate change topics amongst CTTs^22^, our data are able to test for links between funding and specific contrarian claims. Further, we are now able to investigate the types of claims which are linked to funding from concealed donations from “dark money” funders such as Donors Trust/Donors Capital Fund^25,32^, Fig. 4 compares CTT claims with the amount and source of their funding. Brulle^32^ compiled annual funding data of CTTs over the period 2003-2010. We focus our analysis on the association of funding by “key” donors—defined as the ten donors with the highest node degree scores from a network analysis of donors and recipients by Brulle^32^—with CTT climate contrarian communication (Methods). After merging these funding data with our CTT dataset, we were left with 14 observations due to missingness in the Brulle dataset. Figure 4 displays a series of scatterplots which compare the share of funding from these “key” donors with a CTT’s share of category 5 (Fig. 4a), 4 (Fig. 4b), and 1–3 (Fig. 4c) claims. Linear regression results show that the proportion of category 5 and category 1–3 claims are positively associated with the proportion of funding originating from these 10 key donors. Likewise, we find a negative association of category 4 claim prevalence with key donor funding. Figure 4d illustrates the sources of funding for 14 CTTs in our sample. Notably, prominent contrarian CTTs such as the Heartland Institute are heavily dependent upon these key donors and, in particular the “donor-advised” funding flows from Donors Trust and Donors Capital Fund, which ensure anonymous funding to conservative causes^25,32,38^.

## Discussion

Our methodology and findings have significant implications for research on organized climate contrarianism and have the potential to inform practical solutions to identify climate misinformation. Our results offer insights into the ebbs and flows of climate misinformation over two decades, illustrating key differences in claims making by CTTs and contrarian blogs. Figure 2a shows how conservative think tanks were much more likely than blogs to argue that climate change mitigation policies are counterproductive and even harmful. Figure 3a illustrates how this sub-claim consumed over 40% of the claims put forth by CTTs in Q2 2009 and coincided with the drafting and narrow passing of the Waxman-Markey cap-and-trade bill in the U.S. House of Representatives in June 2009. The contrarian blogosphere similarly increased its focus on attacking policy solutions during this time (Fig. 2c). Challenges to climate policy in contrarian blogs have risen steadily over the sample period, with attacks on policy now representing the second most prominent class of claims after the “Science is unreliable”. These findings indicate that moving forward, misinformation is likely to increasingly focus on climate solutions, indicating an important area for future research and public engagement.

Figure 2 also shows one of the novel and consequential insights from this research, revealing how both CTTs and in particular contrarian blogs have invested heavily in propagating narratives that intend to damage the credibility of climate science and climate scientists. This communication strategy includes the use of conspiratorial messaging, as evidenced by the spike in claims calling into question the reliability of the climate science community in 2009, coinciding with the theft of climate scientists’ emails colloquially termed “Climategate”. This contrarian preoccupation with conspiratorial narratives stands in contrast to media articles about climate change where coverage of Climategate dwindled within days^39^. In hindsight, however, this finding should not come as a surprise given that the most common affective response to climate change from those dismissive about climate change is conspiracy theories^40^. This finding is particularly striking given the dearth of research into understanding and countering attacks on science and scientists. While some research has examined attacks on climate scientists^16,41–45^, the bulk of research into climate misinformation has focused on trend, attribution, impact, or solutions contrarianism^10,11,13,14,46,47^. These categories, corresponding to our super-claims “it’s not real”, “it’s not us”, and “it’s not bad”, are the least prevalent forms of climate misinformation. This indicates the need for further research into attacks on climate science and scientists, and the development of educational resources and public communication to counter these efforts.

We also demonstrate the utility of our computational approach by shedding light on the relationship between the claims made by CTTs and donations from core conservative foundations and corporations. Here, we find that money tends to flow to organizations that specialize in challenging the scientific basis of climate science and attacking the integrity of scientists and the broader climate movement. While the current analysis focuses on CTTs, our computational model may be applied to a variety of corpora, including congressional testimonies^17^, traditional media^20^, and social media^42^.

While our project provides a first step in computationally detecting contrarian claims, there are a number of areas that require future research. In this analysis, we show that our model is effective in detecting and categorizing claims in text that is known to come from contrarian sources. However, our algorithm requires further development in order to distinguish between mainstream scientific statements and contrarian statements. Further, our model was generally accurate at categorizing text at the sub-claim level, but we lacked sufficient training data to categorize text at the sub-sub-claim level. Additional training data is required in order to increase the detection resolution of the model. Lastly, other forms of climate misinformation-such as industry-driven greenwashing-are yet to be included in the taxonomy and future research could look to expand the taxonomy.

Nevertheless, our research could help in the effort to develop computer-assisted rebuttals of climate misinformation. There are still many technical challenges towards this goal, requiring the ability to distinguish between contrarian and “mainstream” text on the same topic, and the connection between a framework of claims and refutation content such as the critical thinking-based refutations offered by Cook et al.^28^. Inoculation has been shown to be effective in neutralizing the influence of climate misinformation^3,8^. A holistic “technocognition” solution combining automatic detection, critical thinking deconstruction and inoculating refutations could potentially provide timely responses to rapidly disseminating misinformation online.

## Methods

### Procedure for developing the claims taxonomy

A first draft of the contrarian claims taxonomy was developed based on the list of climate myths at skepticalscience.com. Main categories in this taxonomy reflected the three types of contrarianism (trend, attribution, and impact) outlined in Rahmstorf^10^. The taxonomy was expanded to include policy challenges^14,46^. A fifth category was included to capture consensus claims^48^ and attacks on the integrity of climate science^14^, with the conceptualization of this category clarified over the taxonomy development process.

In addition to including claims referenced in the literature, three authors reviewed thousands of randomly sampled paragraphs to (a) confirm that categories referenced in the literature frequently appear in our corpus of contrarian text and (b) add additional claims as necessary. Specifically, we took small random samples of 50 documents (roughly 800 paragraphs in total) and coded each paragraph down to the sub-sub-claim level shown in Fig. 1. Each annotation was then discussed and the taxonomy and coding instructions were refined in order to reduce ambiguity and increase mutual exclusivity between claims (e.g., added new claims, collapsed multiple claims into a single claim, updated claim wording). This process was repeated until the taxonomy was considered sufficiently stable. A detailed list of the final set of claims and the coding instructions are provided in section S1 of the Supplementary information. An important element of the taxonomy was that the veracity of the claims was not assessed in this analysis-rather, we were documenting claims made in contrarian blogs and conservative websites regardless of their veracity.

Note that while we initially started the taxonomy building process by repeatedly drawing and annotating simple random samples, it became clear that infrequent claims were not sufficiently represented and thus a more targeted sampling scheme was necessary. We carried out a three step procedure to achieve this objective: (1) we started by mapping the general topics reported in Boussalis and Coan^15^ (see Supplementary Table S2) to claims in our taxonomy, (2) we fit Boussalis and Coan’s model to our blog and CTT data, and (3) we over-sampled documents that best matched topics likely to contain contrarian claims.

### Training users to train the machine

#### Pilot coding study

A pilot study to assess the annotation procedure was conducted with undergraduate students $(n=60)$. They scored very low on inter-rater reliability (average kappa = 0.19 across the five categories with highest reliability kappa = 0.3 found for super-claim category 5). Students then submitted an essay, reflecting on their difficulty with the task. The pilot study offered two key insights on the coding procedure. First, we discovered that the design of the coding interface matters: coders performed better if the three level taxonomy was divided into three drop-downs for each level (as opposed to listing all 82 claims in a single drop-down). A web-based, javascript-driven page was programmed to facilitate this multi-step interface. Second, it became clear that a high degree of climate literacy was a requisite skill for reliably performing the coding task. We thus recruited a team of 30 climate-literate volunteers (members of a team who develop and curate scientific content on the SkepticalScience.com website).

#### Annotation procedure

Before they could begin coding, participants watched a training video and performed a training exercise. The script used for the training video and the task employed in the training exercise are provided in the Supplementary information (section S1). Each paragraph was coded independently by at least three coders. Authorship of the paragraph was withheld. Coders coded one (randomly selected) paragraph at a time, assigning a super-claim (and if relevant, a sub-claim and sub-sub-claim) if a contrarian claim appeared in the text. Coders could also flag the paragraph as containing multiple claims, and had the option to choose “Unable to decide” if the text was too difficult to code. If “Unable to decide” was selected, the paragraph went back into the pool of potential paragraphs to annotate. All coders began this process by coding a set of 120 “gold standard” paragraphs, which were subsequently used to assess coder accuracy. These “gold standard” paragraphs consist of 20 paragraphs for each super-claim, as well as 20 paragraphs containing no contrarian claims. A summary of overall coder performance by super-claim is provided in Table 1.

**Table 1:** Average annotator performance by class.

Code	Claim label	Average coder accuracy
0	No claim	0.50
1	Global warming is not happening	0.95
2	Human greenhouse gases are not causing climate change	0.96
3	Climate impacts/global warming is beneficial/not bad	0.97
4	Climate solutions won’t work	0.97
5	Climate movement/science is unreliable	0.86

#### Sampling procedure

Annotation was carried out in two phases. In Phase 1, we coded 31,000 paragraphs randomly selected from our corpus. We found that 93% of the paragraphs did not explicitly make contrarian claims and a number of categories in our taxonomy had too few claims for machine classification. This imbalance is in large part due to our focus on the paragraph-level for annotation, as opposed to document-level, and the fact that articles devote considerable space to background and description. To address the issue of imbalance and weak support for some claims, we carried out a more targeted sampling procedure in Phase 2. First, we used the topic model from Boussalis and Coan^15^ to extract from the corpus 30,000 paragraphs that were more likely to contain contrarian claims. Specifically, we mapped the topic list from Boussalis and Coan to the super-claim categories from our taxonomy (see Supplementary Table S3). This improved balance across classes, with 68% of Phase 2 annotations containing no contrarian claim.

### Classifying contrarian claims: experiments and architecture

The next challenge was to decide on a model suitable for classifying contrarian claims. Note that prior to training extremely short (< 10 words) and extremely long paragraphs (> 2000 characters) were eliminated. Paragraphs consisting of only URLs, scholarly citations, parsing errors, or non-English paragraphs were removed. Paragraphs that were flagged as multiple claims were also eliminated, as were very infrequent classes (i.e., fewer than 50 training samples). As our taxonomy was constructed at the super-, sub-, and sub-sub-claim level, we first needed to decide on an appropriate level of granularity for classification. We decided to focus on the sub-claim level, as this provides considerable detail with respect to contrarian claims, while also ensuring a sufficient level of annotated samples per class to train and test our architecture. Second, we needed to collapse multiple human codings (at least 3 per paragraph) to a single annotation per paragraph. We achieve this objective by using majority rule, where ties were broken randomly. Third, given the thematic and conceptual overlap between sub-claims 5.2 (Movement is unreliable) and 5.3 (Climate is a conspiracy), we collapsed these categories prior to model training. Feedback from our team of annotators and preliminary experiments developing a computational framework on a sample of Phase 1 training data further confirmed this difficulty and thus we do not distinguish between these two sub-claims in this study. Lastly, we needed to address a number of technical challenges associated with the data at hand, namely the need to perform multi-class classification for a large number of classes with extreme class imbalance and noisy label information. We outline our experiments and the steps taken to meet these technical challenges in the remainder of this section.

#### Experiments

Prior to determining our final model architecture, we assessed the performance of a wide range of “shallow” discriminative classifiers and recent “deep” transfer learning architectures^49,50^ in terms of macro-averaged precision, recall, and F1 score. We also experimented with various techniques for class imbalance, including oversampling, weighting^51^, and adjusting our models to use a focal loss function^52^. The results of these experiments are shown in Table 2. In order to provide an accurate assessment of model performance in light of noisy label information and to facilitate comparison across deep and shallow classifiers, we split our annotated paragraphs into a training set $(n = 23,436)$, validation set $(n = 2605)$, and an “error free” test set $(n = 2904)$. To arrive at the “error free” test set, we (1) generated a random sample of annotated paragraphs that matched the class distribution in the training set and (2) re-annotated the test set to fix clear annotation errors. The results in Table 2 suggest that an ensemble of the RoBERTa architecture^50^ and a weighted logistic regression classifier provided the best overall performance. We describe the details of each model in turn.

**Table 2:** Out-of-sample classification performance.

	Validation set (noisy)			Test set (noise free)		
	Precision	Recall	F1	Precision	Recall	F1
Logistic (unweighted)	0.71	0.55	0.62	0.83	0.57	0.68
Logistic (weighted)	0.62	0.68	0.65	0.75	0.70	0.72
SVM (unweighted)	0.66	0.56	0.61	0.77	0.58	0.66
SVM (weighted)	0.60	0.68	0.64	0.74	0.70	0.72
ULMFiT	0.69	0.69	0.69	0.77	0.67	0.72
ULMFiT (weighted)	0.66	0.60	0.62	0.76	0.60	0.65
ULMFiT (over sample)	0.41	0.73	0.50	0.46	0.75	0.55
ULMFiT (focal Loss)	0.66	0.58	0.60	0.73	0.56	0.61
ULMFiT-logistic	0.71	0.70	0.70	0.77	0.72	0.75
ULMFiT-SVM	0.74	0.65	0.70	0.81	0.63	0.71
RoBERTa	0.75	0.77	0.76	0.82	0.75	0.77
RoBERTa-logistic	0.76	0.77	0.76	0.83	0.75	0.79

The table provides macro-averaged precision, recall, and F1 score to compare model fit across “shallow” descriptive classifiers and “deep” transfer learning architectures. *Logistic (Unweighted)*: Logistic regression classifier using TF-IDF weighted features and optimized via grid-search. *Logistic (Weighted)*: Logistic regression classifier using TF-IDF weighted features, weighting for class imbalance, and optimized via grid-search. *SVM (Unweighted)*: A linear support vector machine classifier using TF-IDF weighted features and optimized via grid-search. *SVM (Weighted)*: A linear support vector machine classifier using TF-IDF weighted features, weighting for class imbalance, and optimized via grid-search. *ULMFiT* models: We start with a pre-trained language model which utilizes the Wiki-103 corpus. We then tuned the pre-trained model using 1) our training set $(n = 23,436)$ and a large, random sample $(n = 100,000)$ of unannotated blog and CTT paragraphs. Second, we trained the classification model using the training and validation sets described above. Given observed class imbalances, we examined four variations of the ULMFiT architecture: a model that (1) ignored class imbalance; (2) applies oversampling of each minibatch to adjust for class imbalance; (3) weights the loss function for class imbalance following the “balanced” procedure used in the scikit-learn library; and (4) uses a focal loss function. *RoBERTa* models: See discussion in Methods.

*RoBERTa*. The state-of-the-art pre-trained Transformer Language Model RoBERTA^50^ was employed to train another classifier using the Simple Transformers software package^53^. RoBERTa is an optimized version of the popular BERT language model^54^, which has greatly improved the original model’s performance by optimizing the hyperparameters as well as increasing the training data to five large English-language text corpora^50^. We are using RoBERTa$_{large}$, which was built on the BERT$_{large}$ architecture with 24 layers, 1024 hidden layers, 16 attention-heads and 355M parameters. Our classifier was trained on the training and validation sets (see above), with a range of different hyperparameters. The best performance was achieved with a learning rate of 1e-5, 3 training epochs, a maximum sequence length of 256 and a batch size of 6. To accommodate longer text sequences, a sliding window technique was employed, i.e. longer text sequences were cut into fitting text segments and individually evaluated. To provide the textual context, a stride of 0.6 was defined leading to 40% overlap between the text segments. The severe class imbalance was addressed by specifying “balanced” weights for each class with the scikit-learn library^51^. Experiments with fine-tuning the RoBERTa language model did not improve the results and are, therefore, not further discussed here.

*RoBERTa-Logistic ensemble*. In terms of macro-averaged F1, the standard logistic regression classifier, weighted for class imbalance, was surprisingly competitive with more complex transfer-learning based architectures. Importantly, our experiments suggest that the logistic classifier learns some classes particularly well (e.g., sub-claim 3.2 on “Species/plants/reefs aren’t showing climate impacts yet/are benefiting from climate change”) and, at times, these classes differed from those learned by our best performing RoBERTa model. As such, our final classifier relies on an ensemble of the best performing RoBERTa and logistic classifiers by simply averaging the predicted class probabilities. This ensemble provided a modest gain in performance over RoBERTa alone, with the macro-averaged F1 score on the error-free test set increasing to 0.79. The final F1 score for each super- and sub-claim under consideration is provided in Table 3. The performance is generally good, with the exception of recall for the “Climate policies are harmful” claim. These results, moreover, provide a valuable baseline for future work to improve upon and extend.

**Table 3:** Classification performance by class (claims and sub-claims).

Code		Claim label	Precision	Recall	F1
0	0.0	**No claim**	0.90	0.95	0.93
1		**Global warming is not happening**	0.92	0.80	0.86
	1.1	Ice/permafrost/snow cover isn’t melting	0.92	0.69	0.79
	1.2	We’re heading into an ice age/global cooling	0.73	0.76	0.74
	1.3	Weather is cold/snowing	0.88	0.73	0.80
	1.4	Climate hasn’t warmed/changed over the last (few) decade(s)	0.84	0.67	0.74
	1.6	Sea level rise is exaggerated/not accelerating	0.88	0.92	0.91
	1.7	Extreme weather isn’t increasing/has happened before/isn’t linked to climate change	0.93	0.86	0.90
2		**Human greenhouse gases are not causing climate change**	0.82	0.88	0.85
	2.1	It’s natural cycles/variation	0.82	0.86	0.84
	2.3	There’s no evidence for greenhouse effect/carbon dioxide driving climate change	0.69	0.79	0.73
3		**Climate impacts/global warming is beneficial/not bad**	0.91	0.92	0.91
	3.1	Climate sensitivity is low/negative feedbacks reduce warming	0.82	0.85	0.83
	3.2	Species/plants/reefs aren’t showing climate impacts/are benefiting from climate change	0.81	0.90	0.85
	3.3	CO2 is beneficial/not a pollutant	0.90	0.96	0.93
4		**Climate solutions won’t work**	0.86	0.64	0.74
	4.1	Climate policies (mitigation or adaptation) are harmful	0.70	0.55	0.61
	4.2	Climate policies are ineffective/flawed	0.88	0.44	0.59
	4.4	Clean energy technology/biofuels won’t work	0.72	0.72	0.72
	4.5	People need energy (e.g. from fossil fuels/nuclear)	0.78	0.50	0.61
5		**Climate movement/science is unreliable**	0.82	0.75	0.78
	5.1	Climate-related science is unreliable/uncertain/unsound (data, methods & models)	0.77	0.80	0.77
	5.2	Climate movement is unreliable/alarmist/corrupt	0.78	0.61	0.69

Performance measures are calculated by assessing the final *RoBERTa-Logistic* ensemble classifier using the “error-free” validation set (see Methods).

### Funding data and the selection of “key” donors of contrarian CTTs

For the analysis of the relationship between donor funding and the prevalence of specific contrarian claims generated by CTTs, we relied on financial donation data provided by Brulle^32^ which includes 139 donors and 70 recipients over the period 2003–2010. To narrow the focus of the analysis down to “key” donors, we rely on the results of a network analysis carried out by Brulle on these data. We define “key” donors of contrarian CTTs as the 10 donors with the highest average node degree over the sample period: Donors Trust/Donors Capital Fund $(5.45\%)$, The Lynde and Harry Bradley Foundation, Inc. $(4.70\%)$, Scaife Affiliated Foundations $(4.50\%)$, Koch Affiliated Foundations $(2.96\%)$, John William Pope Foundation $(2.95\%)$, Vanguard Charitable Endowment Program $(2.89\%)$, Searle Freedom Trust $(2.58\%)$, Coors Affiliated Foundations $(2.43\%)$, ExxonMobil Foundation $(2.33\%)$, and Dunn’s Foundation for the Advancement of Right Thinking $(1.45\%)$.

## Supplementary Information

Supplementary Information.

## Data Availability

The analysis data is available at https://socialanalytics.ex.ac.uk/cards/data.zip. The classifiers are available at https://socialanalytics.ex.ac.uk/cards/models.zip.

## Code availability

The analysis code is available at https://github.com/traviscoan/cards.
