# Supplementary material for: Computer-assisted classification of contrarian claims about climate change
Source: Sci Rep. 2021 Nov 16;11:22320. doi: 10.1038/s41598-021-01714-4 (PMC8595491; doi:10.1038/s41598-021-01714-4)
Supplement: Supplementary file 1 — Supplementary Information. [file 41598_2021_1714_MOESM1_ESM.pdf]

Supplementary information

Computer-assisted classification of contrarian claims about climate change

Travis G. Coan[1], Constantine Boussalis[2], John Cook[3,4,*], and Mirjam O. Nanko[1]

[1]Department of Politics and the Exeter Q-Step Centre, University of Exeter, United Kingdom.

[2]Department of Political Science, Trinity College Dublin, Ireland.

[3]Monash Climate Change Communication Research Hub, Monash University, Australia.

[4]Center for Climate Change Communication, George Mason University, USA.

[*]Corresponding author email: john.cook@monash.edu

S1 Supplementary Methods 3

S1.1 Training materials 3

S1.1.1 Training text 3

S1.1.2 Training exercise 7

Table S1: Paragraphs used in training exercise 7

S1.1.3 Code book 8

S1.1.4 Taxonomy of Contrarian Claims 16

Table S2: Contrarian claims 16

S1.2 Mapping topics to super-claims 19

Table S3: Boussalis & Coan (2016) topics mapped to super-claims 19

S2 Supplementary Data and Results 21

Figure S1: Total monthly number of CTT documents and blog posts 21

Table S4: Corpus of conservative think tank climate-related documents (N = 115,537) 22

Table S5: Corpus of climate change-related blog posts (N = 139,912) 23

Table S6: Linear regression results of donations from “key” donors and claim prevalence 24

S1 Supplementary Methods

S1.1 Training materials

S1.1.1 Training text

This is the transcript for the training video that all coders were required to view before coding:

CARDS: Computer Assisted Recognition of Denial and Skepticism, is a machine learning project. Our aim is to train a computer to automatically detect and categorize misinformation about climate change. The end goal is that a computer can look at some text and successfully identify any climate misinformation - and even identify specific denialist claims. If successful, this will enable us to travel back in time and build a history of climate misinformation, including when myths originated and how they've evolved over time. It will also enable us to spot new publishing of denialist claims in real-time.

We train the computer by reading climate denialist articles, and matching paragraphs to specific denialist claims. Each time we match some text to a particular claim, we train the computer to get better at automatically detecting claims.

In order to help you match text to denialist claims in a consistent, reliable way, I need to explain two things. First, I'll introduce our taxonomy of denialist claims - giving you an overview of the claims you're likely to encounter. Second, I'll take you through the step-by-step process to go through when looking at each paragraph.

Taxonomy of denialist claims

Let's start with a quick, 10-word introduction to climate change. There are 5 key facts that summarize everything you need to know about climate change. And they are:

1. It's real
2. It's us
3. It's bad
4. There's hope
5. Experts agree

We've developed a taxonomy of denialist claims that aim to cast doubt on climate science. Climate misinformation can be broken into five main categories, which we call super-claims. They're the opposite of the five climate beliefs: it's not real, it's not us, it's not bad, experts are unreliable and there's no hope.

1. It's not real
2. It's not us
3. It's not bad
4. Experts are unreliable

5. There’s no hope

In our taxonomy, we used slightly different wording to describe these five super-claims. They are:

1. Global warming is not happening
2. Human greenhouse gases are not causing global warming
3. Climate impacts will not be bad
4. Climate solutions won’t work
5. The climate movement/science is unreliable

Under each super-claim are sub-claims. For example, under the super-claim “global warming isn’t happening” is the sub-claim “Weather is cold or snowing”.

Under some of the sub-claims, there are sub-sub-claims. For example, under the sub-claim “Ice isn’t melting”, you’ll find the sub-sub-claims “Antarctica is cooling” or “Greenland is gaining ice.”

The next stage of denial after “it’s not happening” is “okay, it is happening, but it’s not us causing it”. This usually involves blaming global warming on natural causes, like the sun... volcanoes... or the ocean. This includes claims that minimize the human contribution. For example, saying humans aren’t causing most of global warming.

Another popular argument against human-caused global warming is to say “climate changed in the past before humans... so it must be natural now.” Whenever you see claims about climate changing or being warmer in the past, whether it be the early 20th century or the Earth’s deep past, you’ll find this sub-sub-claim under the sub-claim “it’s natural cycles.”

The next stage of denial is “okay, it’s happening, we’re causing it, but the impacts aren’t that bad.” For example, one of the most popular ways of downplaying climate impacts is to say, “CO2 emissions are actually a good thing. Plants need CO2 to grow. It’s plant food!”

Sometimes it’s hard to distinguish between a claim that global warming isn’t happening and a claim that climate impacts aren’t happening. The difference is that impacts are about the effects that global warming causes rather than the mere existence of global warming. For example, if you see terms like “dangerous” or “catastrophic”, then that’s about impacts, not about the existence of warming.

Another claim that downplays impacts is that climate sensitivity is low. This includes claims that negative feedbacks will reduce the climate response to global warming.

The final stage of denial is “okay, it’s happening, it’s us, it’s bad, but there’s nothing we can do about it.” This is about the solutions to climate change, whether it be policies like carbon pricing, or technologies like solar and wind.

Note the distinction between climate policy and clean energy technology. Claims about specific technologies such as wind turbines and solar PV come under the clean energy sub-claim. But more general claims about climate solutions that aren’t technology-related come under either climate policies are harmful or climate policies are ineffective.

The last category involves attacks against climate science, or people involved in climate change. Note that the super-claim uses the general term climate movement. This is because denialist attacks aren’t

just directed at climate scientists. They can also target politicians, environmentalists or media outlets. So when you read an ad hominem attack on a person or group, look to see whether it matches one of our sub-groups. If not, go with the broader category 5.2, covering the climate movement in general.

Another important distinction is between bias versus deception. Claims that scientists are deliberately deceiving people or engaged in a conspiracy comes under the conspiracy sub-claim. Less extreme claims that scientists are biased or wrong or unreliable come under 5.2: climate movement is alarmist/wrong/political/biased.

We've also have a visual form of the taxonomy in PDF form, created by our own Baerbel Winkler. You can find this PDF via the navigation links. We recommend you have a print-out of the taxonomy beside your computer as you code articles.

Coding flowchart

We've made a coding flow-chart, which gives you the step-by-step process to go through when you're coding denialist text. The point of the flow-chart is to make this process as consistent as possible. For the rest of this video, I'll take you through the steps of the flow-chart and show some examples along the way.

Some of the articles you'll encounter won't be climate related. We scraped from climate denialist blog posts and conservative think-tank websites, and not all of their articles are climate-related. So if the article is obviously not climate-related, just scroll to the bottom of the form and hit submit. Even for climate-related articles, you'll find most paragraphs won't match a specific claim so don't feel pressured to select a claim.

To train a computer to accurately identify denialist claims, you want to see two types of keywords. The first type are climate-related keywords. For example, imagine a paragraph about global temperature that has climate-related keywords like "surface temperature" or "thermometer measurements". It might be text that's simply about temperature. But we want to accurately detect denialist claims. So it's not enough to have just climate keywords like temperature or thermometers.

The second type of keywords we need to see are denialist keywords. For example, we might be looking for keywords that express the denialist claim that the temperatures are not warming. As well as climate keywords about temperature, you're also looking for denialist keywords such as "not warming" or "lack of warming". It's only when you have both types of keywords: climate keywords and denialist keywords, that you have a denialist claim. If you don't see both types of keywords, then don't select a claim in the dropdown. You can do this either by selecting "no match" or just do nothing.

You code each article one paragraph at a time. To the right of each paragraph is a single drop-down listing the five super-claims. As you read each paragraph, if you see a super-claim, select it in the drop-down. If you do select a super-claim, then a second drop-down will appear with sub-claims. If you see a sub-claim in the paragraph, select that in the sub-claim drop-down - noting that there may not be any sub-claim that fits. Sometimes, there are sub-sub-claims underneath the sub-claim you just selected. In those cases, a third drop-down will appear listing the sub-sub-claims. If you see a sub-sub-claim in the paragraph text, select that also in the drop-down.

If the paragraph includes only one denialist claim in the paragraph, then select the claim in the drop-down.

If the paragraph contains more than one claim, then we need to be extra careful. Remember we're training the computer to associate certain words with certain claims. We don't want to confuse it - a paragraph needs to be clearly linked to a single claim. So if a paragraph has multiple claims, only select a claim if the paragraph is mostly about a single claim, with most of the keywords in the paragraph about that claim.

For example, here's a paragraph where the bulk of the text is about China or India. There's a second, brief claim about cheap electricity. But while the paragraph has multiple claims, most of the text is about the China or India claim.

If the paragraph has multiple claims but no clear, dominant claim, then select "Multiple Claims" and move onto the next paragraph.

If you select a super-claim, then the sub-claim drop-down will appear. Repeat the same flowchart process for the sub-claim, remembering that sometimes no sub-claim is appropriate. If you select a sub-claim, then sometimes a sub-sub-claim drop-down will appear. Again, repeat the flowchart process for the sub-sub-claim.

Now let me take you through a few issues you might encounter. Here's a tricky one. Sometimes you'll see multiple claims that fall under the same super-claim. In those cases, do select the common super-claim. For example, this paragraph contains two claims - climate policy will increase prices... and green energy is unreliable. At a super-claim level, both claims are "climate solutions won't work." So go ahead and select that super-claim. But at the sub-claim level, it's about two different sub-claims: "climate policies are harmful" and "clean energy technology won't work." So at the sub-claim level, select multiple claims.

When the text includes accusations of deception, opt for 5.3 Climate change is a conspiracy. We distinguish between claims of bias under 5.2, which are about being mistaken or unreliable, and accusations of intentional deception under 5.3.

To keep our taxonomy simple, we didn't include all the claims you're likely to encounter. Nevertheless, any claim you encounter should come under the umbrella of claims in our taxonomy. For example, one claim is Extreme weather isn't increasing. This claim covers all types of extreme weather. For example, flooding, heat waves, drought, hurricanes, tornadoes and wildfires. In addition, the extreme weather claim can take several different forms. As well as the most straightforward claim that "extreme weather isn't increasing", there are two other extreme-weather related claims. They are "extreme weather isn't linked to global warming" and "extreme weather has happened in the past". If you see either type of claim, select 1.7 Extreme weather isn't increasing.

The system keeps track of how long you spend coding each article. As soon as the page loads, the clock starts. As soon as you hit submit, the clock ends. So only code one article at a time and do a single article all in one sitting. Ideally, don't load an article, go off and do something else like make a cup of coffee, then come back to it. Make your coffee first, come back to your computer, click on the "Start coding!" link to reload the page and restart the clock. Knowing how long it takes us all to code each article will be useful data. Some of our fans will be interested in this data too.

Most paragraphs won't match a specific claim so don't feel pressure to select a claim. Regardless of whether you select any claims or not, hitting submit will add those paragraphs to your tally for the leaderboard. If you're not sure, don't select anything. A false negative, where you don't select a claim, won't have any impact on our overall reliability or how good we train the computer. But a false positive will reduce reliability and make training the computer more difficult. So only select a claim if you're sure. If in doubt, leave it out. If uncertain, close the curtain. If it doesn't fit, you must acquit. You can either do nothing or choose "no match". It's the same either way. No claim is selected.

Sometimes our scraping of articles had technical problems. Some articles got broken up into fragments, others got bundled into one long paragraph. In all cases, follow the same process. Look for climate keywords plus denialist keywords. Look for a single denialist claim. Usually very long paragraphs will contain multiple claims so they're unlikely to contain a single claim.

At the bottom of each article is a feedback text-box. Only enter something in here if you feel the instructions need clarifying or the methodology needs updating. Your feedback will be immediately emailed to me and we'll assess whether our instructions or methodology need updating based on your feedback.

Hopefully these instructions should tell you all you need to start coding. A transcript of this video is also shown below the video. If you have any further questions, first consult the FAQ. If you have still have any questions, either ask them on the Skeptical Science forum or email me directly.

Finally, we've created a CARDS T-shirt - we'll be giving free CARDS T-shirts to the top coders. We don't know exactly how many T-shirts we'll give out yet. Quality as well as quantity is important - we'll also be looking at inter-rater reliability. So make sure you read the text carefully as you do your coding - reliability is crucially important to this project being successful.

Good luck and thank you for helping us to identify climate misinformation.

S1.1.2 Training exercise

Before being able to code, participants performed the following training exercise, coding this selection of paragraphs. If they coded the paragraph incorrectly, they were displayed an error message explaining how their coding was incorrect.

Table S1: Paragraphs used in training exercise

Denialist Paragraph	Correct Claim	Instructional Feedback
What we are experiencing is outside of anything humans have seen on our planet and the only explanation that makes any real sense is that it is due to human actions.	No claim	For expressions of mainstream climate science, select No Match.
There have only been three recorded hurricanes during May in the Atlantic Basin. They occurred in 1908, 1951 and 1970, including this hurricane which struck the US during the last week of May, 1908.	1.7. Extreme weather isn't increasing/has happened before/isn't linked to climate change.	Extreme weather keywords plus denialist keywords, should be coded as 1.7. Denialist keywords include references to extremes in the past, extremes not increasing or extremes not linked to climate change.

Another record was broken in Vladivostok, which registered the lowest absolute minimum air temperature in more than 130-year history of weather observations.	1.3. Weather is cold/snowing.	Talk of cold weather or snow should be coded as 1.3.
Either an RES or CES would also raise the average cost of generating electricity in the United States because, in the absence of the standard, regulators and generators would generally choose the lowest-cost method of producing electricity.	4.1.1. Climate policy will increase costs/harm economy/kill jobs.	Claims that climate policy will increase costs or harm the economy should be coded 4.1.1.
Didn't Hansen predict catastrophic sea level rise and increased hurricanes within 20 years in 1988? Doesn't anyone look at his record of prediction? Pushing the scare seems to be the thing.	5.2.5. Scientists/academics are alarmist/wrong/ political/biased.	If the text insults people or groups who support climate science/action, then this will come under sub-claim 5.2. If the text specifies a person/group who is specifically media, politicians, environmentalists or scientist, then go to the sub-sub-claim level.
Over the last 16 years, global average temperature, as measured by both thermometers and satellite sensors, has displayed no statistically significant warming.	1.4. Climate hasn't warmed/changed over the last (few) decade(s).	The paragraph contains both climate keywords and contrarian keywords.
The IPCC argues that feedbacks from increased water evaporation will lead to enhanced warming. This is not observed in those regions most effected by water vapour. In fact the opposite seems to be the case implying negative feedback.	3.1. Climate sensitivity is low/negative feedbacks reduce warming.	Arguments for negative feedback (or low positive feedback) imply climate sensitivity is low.
Sea surface temperature records indicate El Nino and La Nina events are responsible for the warming of global sea surface temperature anomalies over the past 30 years, not manmade greenhouse gases.	2.1.3. It's the ocean/internal variability.	Blaming global warming on ocean cycles like El Nino or La Nina should be coded 2.1.3.
Both summer and winter temperatures over much of the Medieval Warm Period throughout many parts of Finland were significantly warmer than they are currently. In this part of the world, therefore, the climate-alarmist claim that current temperatures are unprecedented over the past one to two millennia rings rather hollow.	2.1.5. Climate has changed naturally/been warm in the past.	Reference to past climate change or warmer periods before the modern global warming period should be coded 2.1.4.

S1.1.3 Code book

Super-claim: highest level claim (not happening, not bad, etc), also known as logic stream

Sub-claim: second level claim (e.g., 1.1 Ice isn't melting)

Sub-sub-claim: third level claim (e.g., 1.1.1 Antarctica is cooling)

Keyword: words that are uniquely linked to a given claim; not a stop-word

1. If parsing issues, ***flag as "bad parse" and code blank***.

2. Else if paragraph is an excerpt from warmist, ***code blank***.
3. Else if text contains text that is explicitly about *whole* claim/s:
    3.1. If text contains one claim, ***code claim***.
    3.2. Else if text contains more than one claim:
        3.2.1. If "central claim" is clear (clear plurality of keywords), ***code central claim & flag multi***.
        3.2.2. Else if claims are direct ancestor/descendant, ***code lowest level claim & flag multi***.
        3.2.3. Else if common ancestor exists (e.g., under same "super parent"), ***code ancestor & flag multi***.
        3.2.4. Else, ***code blank & flag multi***.
4. If text may imply a claim:
    4.1. If claim isn't explicitly made elsewhere in the article, ***code blank***.
    4.2. Else if claim is explicitly made elsewhere keywords that are uniquely linked to the implied claim (including contrarian/negative keywords), ***code the context claim***.
    4.3. Else, ***code blank***.

Frequently Asked Questions

Q: Why am I seeing a lot of the articles not about climate?

A: We scraped ~250,000 blog posts from 50 denialist blogs. It wasn't practical to manually remove non-climate related posts. So you will see non-climate related posts, or climate-related blog posts with no specific denialist claims. Note that in phase 2, only 7% of paragraphs were coded with a denialist claim (in phase 3, we were more selective with paragraphs so 30% of the paragraphs were coded with a denialist claim). If the paragraph is off-topic or doesn't contain a claim, just hit submit without selecting anything in the dropdown.

Q: Does it matter whether I select 'no match' in the drop-down or don't do anything?

A: It makes no difference whatsoever. Either way, the paragraph gets coded as 0 (e.g., not containing a claim).

Q: What do we do with what looks like a bad Google translate?

A: In these cases, don't select a claim as we don't want the machine associating gobbledegook with denialist claims.

Q: When do I enter something in the comment box?

A: The comment text box is for questions and comments. E.g., if you're a little fuzzy on how to code some text (even after reading this whole FAQ), put your question in the comment box and it will get emailed immediately to John Cook, who will respond ASAP. You can also flag scholarly citations with the word "cite", and paragraphs that were parsed badly (e.g., sentence fragment) with "parse".

Q: How does it time how long it takes to code an article?

A: The clock starts as soon as the coding form loads. The clock stops once you hit submit. So after hitting submit, a new article will immediately load and the clock starts again. But you don't have to code the next article. You can always restart the clock by clicking the navigation link “Start coding!”, which will load a new coding form and restart the clock.

Q: How do we code pro-science articles that state contrarian arguments in order to rebut them?

A: If a pro-science paragraph contains climate keywords and denialist keywords associated with a specific denialist claim, then code it. It's about the keywords, not the intent. Note that separate to this project, we’ve also scraped tens of thousands of blog posts from “convinced” climate blogs. So future analysis will be about training a machine to distinguish between convinced and denialist text. But one step at a time...

Q: What if the text contains claims that support mainstream climate science, or alarmist myths?

A: Our research focus is on denialist myths. We’ll be content analysing convinced text in follow-up research. So we’re only looking for text that includes climate keywords and denialist keywords associated with a specific denialist claim. So code it as not containing a denialist claim.

Q: Does text "X" belong to claim "Y"?

A: Here is some guidance on how to code specific types of claims:

1.1. Ice isn’t melting/snow cover isn't vanishing

- Includes "permafrost isn’t melting"

1.1.3. Arctic sea ice isn’t vanishing

- Includes "Arctic sea ice is drifting/pushed by winds"

1.1.4. Glaciers aren’t vanishing

- Includes "glaciers aren't melting, it's getting drier"

1.2. We’re heading into an ice age/global cooling

- Includes "no trend in future"
- Includes "sea levels will fall in the future"

1.4. Climate It hasn’t warmed/changed over the last (few) decade(s)

- Includes explanations of what caused the hiatus
- Includes cooling

1.5. Oceans are cooling/not warming

- Includes "heat isn't being transferred to the deep ocean"

1.6. Sea level rise is exaggerated/not accelerating

- Includes "SLR is caused by land subsidence/isostatic adjustments"
- Includes "melting sea ice doesn’t contribute to SLR"
- Includes "islands are not sinking"
- Includes "SLR is cancelled out by isostatic rebounding"
- Includes "SLR is due to groundwater depletion"

1.7. Extreme weather isn’t increasing/has happened before/isn’t linked to climate change

- Includes "increases in extreme weather is due to better observations/more populated areas"
- Includes "damages/deaths from extreme weather aren’t increasing"
- Includes "extreme weather linked to non-climate change phenomena (like ENSO)"
- Includes bushfires/wildfires

2.1. It’s natural cycles/variation

- Includes clouds (if not mentioning 2.1.1. cosmic rays)
- Includes "it's gravity/pressure"

2.1.1. It’s the sun/cosmic rays/astronomical

- Includes Milankovitch cycles
- Includes "other planets are warming"

2.1.2. It’s geological (includes volcanoes)

- Includes heat sources within the Earth
- Includes "volcanoes are melting ice"

- Includes changing Earth’s magnetics
- Includes "removal of cooling volcanic eruptions caused warming"

2.1.3. It’s the ocean/internal variability

- Includes "ice melt is caused by ocean cycles"

2.1.4. Climate has changed naturally/been warm in the past

- Includes "Medieval Warm Period (MWP) was warmer/global"
- Includes "it warmed in the early 20th century"
- Includes "we’re coming out of the Little Ice Age"
- Includes "glaciers have changed in the past"
- Includes "sea levels have changed/been higher in the past"
- Includes "Greenland was green"

2.2. It’s non-greenhouse gas human climate forcings (aerosols, land use)

- Land use includes deforestation
- Includes "CFCs causing ozone depletion"
- Includes "ozone depletion is causing global warming"
- Includes brown carbon, soot (e.g., soot causing ice melt)
- Includes "absence of aerosols/dimming is causing warming"
- Includes "it's waste heat from industry/fossil fuel burning"

2.3. There’s no evidence for greenhouse effect

- Includes "greenhouse effect doesn’t exist/contradicts the 2nd law of thermodynamics"
- Includes "CO2 has cooling effect"
- Includes "greenhouse effect/infrared can't warm oceans"
- Includes minimizing human contribution to less than half of observed warming
- Includes "anthropogenic greenhouse effect is cancelled out by aerosols"
- Includes "warming caused by convection/convection, not radiation"
- Includes "outgoing longwave radiation is increasing"

2.3.2. Greenhouse effect is saturated

- Includes logarithmic relationship between CO2 and warming

2.3.3. Carbon dioxide lags/not correlated with climate change

- Includes "oceans are giving up co2 in response to warming"
- Includes "temperature drives CO2"
- Includes "it cooled mid-20th-Century while CO2 increased"
- Includes "warming causes CO2 to rise"

2.3.5. There’s no tropospheric hot spot

- Includes "hot spot is unique signature of GHG warming"

2.5. Human CO2 emissions are miniscule/not raising atmospheric CO2

- Includes "CO2 has a short residence time"
- Includes large CO2 emissions from fire
- Includes CO2 emissions from natural sources (e.g., ocean)
- Includes "CO2 is not going to reach dangerous levels"
- Includes "CO2 measurements were higher in 19th/early 20th century"
- Includes "CO2 has been highly variable in the past"

3.0. Climate impacts/global warming is beneficial/not bad

- Includes "extinction is natural"
- Includes claims about optimal/ideal temperature/climate
- Includes "cold is bad" (but if gets specific about cold deaths, then code as 3.6)

3.1. Climate sensitivity is low/negative feedbacks reduce warming

- Includes claims that atmospheric water vapor is decreasing
- Includes "no runaway warming" or "no tipping point"
- Increasing natural CO2 absorption is a negative feedback

3.2. Species/plants/reefs aren’t showing climate impacts yet/are benefiting from climate change

- Includes "species are being impacted by non-climate change factors"

3.2.3. Ocean acidification/impacts on coral aren’t serious

- Includes coral impacts from warmer waters/bleaching as well as acidification
- Includes corals rising to counter SLR
- Includes "Coral impacts aren't caused by climate change"

3.6. Climate change doesn’t negatively impact health

- Includes "cold kills more than heat"
- Includes "global warming isn’t helping spread disease"

4.1. Climate policies (mitigation or adaptation) are harmful

- Includes "climate policy will/has caused power outages"

4.1.1. Climate policy will increase costs/harm economy/kill jobs

- Includes "climate policy is expensive"

4.1.5. Climate policy limits liberty/freedom/capitalism

- Includes "wealth redistribution scheme"

- Includes linking climate to communism
- Includes claims of government control

4.2. Climate policies are ineffective/flawed

- Includes "emissions didn't drop due to climate policy but because of other factors"
- Includes "policy can be gamed/manipulated/scammed"

4.2.2. Markets/private sector are economically more efficient than government policies

- Includes "cap and trade is better than carbon tax/regulation"
- Includes claims attacking renewable subsidies

4.2.3. Climate policy will make negligible difference to climate change

- Includes "policy will cause negligible reduction in emissions"

4.2.5. Better to adapt/geoengineer/increase resiliency

- Includes "Future generations will be richer and better able to adapt"

4.3. It’s too hard to solve

- Includes "it’s too late to fix climate change"
- Includes "it would continue to warm even if we stopped emissions"

4.4.1. Clean energy/biofuels are too expensive/unreliable/counterproductive/harmful

- Includes ethanol
- Includes batteries
- Includes "local residents don’t like wind farms"
- Includes "renewables can't survive without subsidies"
- Includes "wind turbines disrupt radar"

4.4.2. Carbon Capture & Sequestration (CCS) is unproven/expensive

- Includes "CCS isn't competitive"
- Includes "CCS is ineffective at reducing emissions"

4.5.2. Fossil fuels are cheap/good/safe for society/economy/environment

- Includes "fracking is safe"

4.5.3. Nuclear power is safe/good for society/economy/environment

- Includes "nuclear power is cheap"

5.1. Climate-related science is uncertain/unsound/unreliable

- Includes "predicted ice age in 1970s"
- Includes attack on other climate data (besides temperature record, proxy data and climate models) such as sea level.
- Includes uncertainty about future climate tech/policy predictions
- Includes "everything is used to prove GW/AGW"
- Includes "need more data to properly understand climate"

5.1.1. There’s no scientific consensus on climate/the science isn’t settled

- Includes "science isn't done by consensus"
- Includes "consensus isn’t valid"
- Includes "consensuses have been overturned in the past"

5.1.3. Temperature record is unreliable

- Includes "it's urban heat island effect"

5.1.4. Models are wrong/unreliable/uncertain

- Includes "climate models can't even predict the weather"
- Includes integrated assessment models (IAMs)
- Includes "climate models aren't falsifiable"

5.2. Climate movement is alarmist/wrong/political/biased/hypocritical (people or groups)

- Includes "warmists blame everything on global warming" (can also be applied to any of the 5.2 sub-sub-claims)

5.2.2. Media (including bloggers) is alarmist/wrong/political/biased

- Includes climate blogs (including Skeptical Science)

5.2.3. Politicians/government/UN are alarmist/wrong/political/biased

- Includes EPA, UN, UNFCCC

5.2.4. Environmentalists are alarmist/wrong/political/biased

- Includes Al Gore

5.2.5. Scientists are alarmist/wrong/political/biased

- Includes science-based organizations such as IPCC, UKMO, NSIDC, NASA, NOAA
- Includes scholarly journals/publishers
- Includes social scientists/philosophers/economists who support climate science/action

5.3.2. Climate science is a hoax/scam/conspiracy/secretive/money-motivated (includes climategate)

- Includes accusations of data tampering
- Includes claims about climategate “whitewash” investigations
- Includes claims of withholding data (secretive)
- Includes "scientists are in it for the money

S1.1.4 Taxonomy of Contrarian Claims

These claims are visualized in Figure 1.

Table S2: Contrarian claims

Code	Identifier	Claim
1	1	Global warming is not happening
1.1	6	Ice/permafrost/snow cover isn't melting
1.1.1	7	Antarctica is gaining ice/not warming
1.1.2	8	Greenland is gaining ice/not melting
1.1.3	9	Arctic sea ice isn't vanishing
1.1.4	10	Glaciers aren't vanishing
1.2	11	We're heading into an ice age/global cooling
1.3	12	Weather is cold/snowing
1.4	13	Climate hasn't warmed/changed over the last (few) decade(s)
1.5	14	Oceans are cooling/not warming
1.6	15	Sea level rise is exaggerated/not accelerating
1.7	16	Extreme weather isn't increasing/has happened before/isn't linked to climate change
1.8	17	They changed the name from 'global warming' to 'climate change'
2	2	Human greenhouse gases are not causing climate change
2.1	18	It's natural cycles/variation
2.1.1	19	It's the sun/cosmic rays/astronomical
2.1.2	20	It's geological (includes volcanoes)
2.1.3	21	It's the ocean/internal variability
2.1.4	22	Climate has changed naturally/been warm in the past
2.1.5	23	Human CO2 emissions are tiny compared to natural CO2 emission
2.2	24	It's non-greenhouse gas human climate forcings (aerosols, land use)
2.3	25	There's no evidence for greenhouse effect/carbon dioxide driving climate change
2.3.1	26	Carbon dioxide is just a trace gas
2.3.2	27	Greenhouse effect is saturated/logarithmic
2.3.3	28	Carbon dioxide lags/not correlated with climate change
2.3.4	29	Water vapor is the most powerful greenhouse gas

2.3.5	30	There's no tropospheric hot spot
2.3.6	75	CO2 was higher in the past
2.4	76	CO2 is not rising/ocean pH is not falling
2.5	78	Human CO2 emissions are miniscule/not raising atmospheric CO2
3	3	Climate impacts/global warming is beneficial/not bad
3.1	31	Climate sensitivity is low/negative feedbacks reduce warming
3.2	32	Species/plants/reefs aren't showing climate impacts yet/are benefiting from climate change
3.2.1	33	Species can adapt to global warming
3.2.2	34	Polar bears are not in danger from climate change
3.2.3	77	Ocean acidification/coral impacts aren't serious
3.3	35	CO2 is beneficial/not a pollutant
3.3.1	36	CO2 is plant food
3.4	37	It's only a few degrees (or less)
3.5	38	Climate change does not contribute to human conflict/threaten national security
3.6	39	Climate change doesn't negatively impact health
4	4	Climate solutions won't work
4.1	40	Climate policies (mitigation or adaptation) are harmful
4.1.1	41	Climate policy will increase costs/harm economy/kill jobs
4.1.2	42	Proposed action would weaken national security/national sovereignty/cause conflict
4.1.3	43	Proposed action would actually harm the environment and species
4.1.4	44	Future generations will be richer and better able to adapt
4.1.5	45	Climate policy limits liberty/freedom/capitalism
4.2	46	Climate policies are ineffective/flawed
4.2.1	47	Clean energy/green jobs/businesses won't work
4.2.2	48	Markets/private sector are economically more efficient than government policies
4.2.3	49	Climate policy will make negligible difference to climate change
4.2.4	50	A single country/region only contributes a small % of global emissions
4.2.5	51	Better to adapt/geoengineer/increase resiliency
4.2.6	52	Climate action is pointless because of China/India/other countries' emissions
4.2.7	80	We should invest in technology/reduce poverty/disease first
4.3	53	It's too hard to solve
4.3.1	54	Climate policy is politically/legally/economically/technically too difficult
4.3.2	79	Media/public support/acceptance is low/decreasing
4.4	55	Clean energy technology/biofuels won't work
4.4.1	56	Clean energy/biofuels are too expensive/unreliable/counterproductive/harmful
4.4.2	57	Carbon Capture & Sequestration (CCS) is unproven/expensive
4.5	58	People need energy (e.g., from fossil fuels/nuclear)

4.5.1	81	Fossil fuel reserves are plentiful
4.5.2	82	Fossil fuels are cheap/good/safe for society/economy/environment
4.5.3	83	Nuclear power is safe/good for society/economy/environment
5	5	Climate movement/science is unreliable
5.1	59	Climate-related science is uncertain/unsound/unreliable (data, methods & models)
5.1.1	60	There's no scientific consensus on climate/the science isn't settled
5.1.2	61	Proxy data is unreliable (includes hockey stick)
5.1.3	62	Temperature record is unreliable
5.1.4	63	Models are wrong/unreliable/uncertain
5.2	64	Climate movement is alarmist/wrong/political/biased/hypocritical (people or groups)
5.2.1	65	Climate movement is religion
5.2.2	66	Media (including bloggers) is alarmist/wrong/political/biased
5.2.3	67	Politicians/government/UN are alarmist/wrong/political/biased
5.2.4	68	Environmentalists are alarmist/wrong/political/biased
5.2.5	69	Scientists/academics are alarmist/wrong/political/biased
5.3	70	Climate change (science or policy) is a conspiracy (deception)
5.3.1	71	Climate policy/renewables is a hoax/scam/conspiracy/secretive
5.3.2	72	Climate science is a hoax/scam/conspiracy/secretive/money-motivated (includes climategate)

S1.2 Mapping topics to super-claims

Table S3: Boussalis & Coan (2016) topics mapped to super-claims

Topic No.	Topic from B&C (2016)	Super-claim Category
topic1	Climate sensitivity to CO2	2
topic2	Fossil fuel production	
topic3	Sea level rise	1
topic4	No scientific consensus	5
topic5	Long-term climate trends	1
topic6	Public opinion	
topic7	US politics	
topic8	Renewable energy	4
topic9	Costs of govt. intervention	4
topic10	Environmentalism	
topic11	Climate models	5
topic12	Solar forcing & cloud models	5
topic13	Temperature station data	5
topic14	Scientific misconduct	5
topic15	Govt. agencies	4
topic16	Alarmism	5
topic17	International relations	4
topic18	Agricultural industry	4
topic19	Human health	
topic20	Corporations & the environment	4
topic21	Urban economics	4
topic22	Reuse & recycle	4
topic23	Nuclear power	4
topic24	Green jobs	4
topic25	Economic impact of climate policy	4
topic26	Monckton	
topic27	IPCC integrity	5
topic28	Storms	3
topic29	Emissions reduction	4
topic30	Plant impacts	3
topic31	International trade and development	
topic32	Tax & spend	
topic33	Conservation	

topic34	Forest impacts	3
topic35	Cap & trade	4
topic36	Public transportation	
topic37	Climate adaptation	4
topic38	EPA	4
topic39	Law	4
topic40	State climate reports	
topic41	State climate policy	
topic42	Acidification	3
topic43	Disaster costs	
topic44	International climate agreements	4
topic45	Pollution	
topic46	Endangered species	3
topic47	Automobile fuel standards	4

S2 Supplementary Data and Results

Figure S1: Total monthly number of CTT documents and blog posts (note that the y-axes are on different scales)

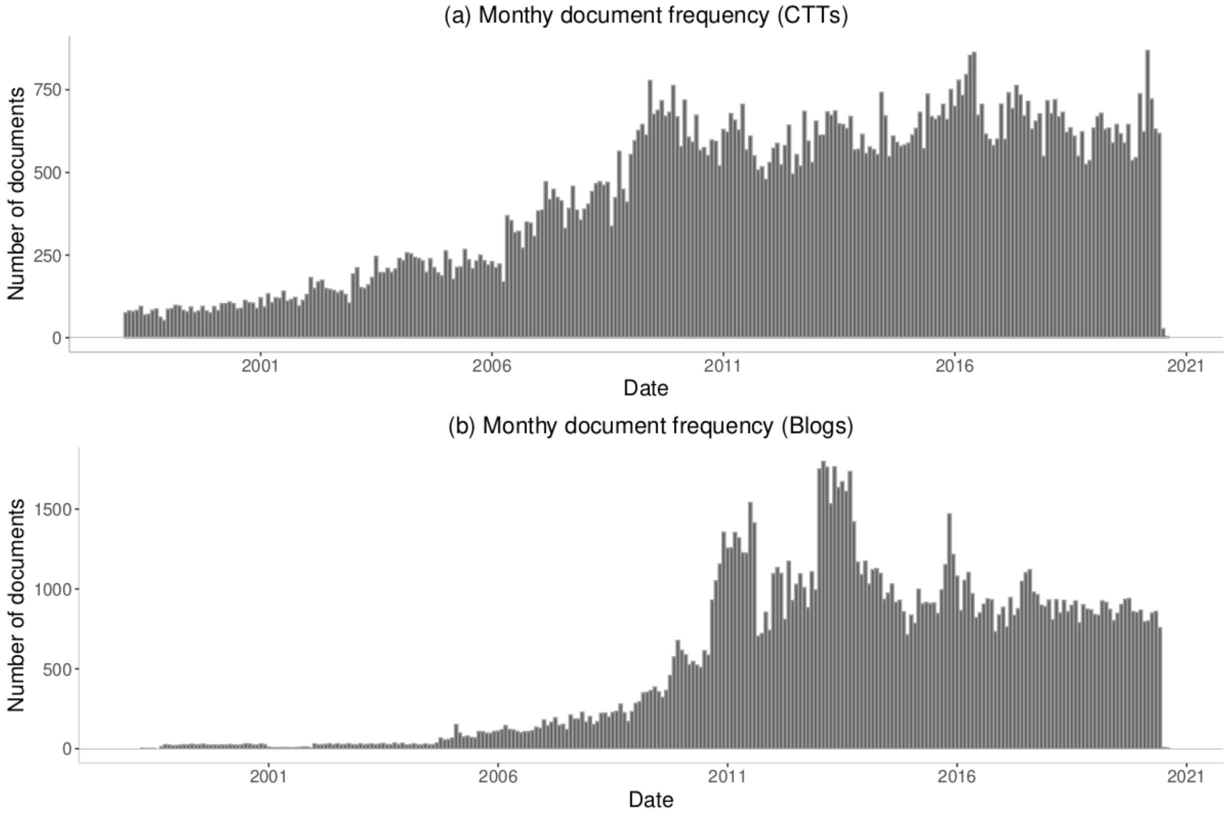

Table S4: Corpus of conservative think tank climate-related documents (N = 115,537)

CTT Name	Country	Language	Year min	Year max	Docs
American Policy Center	USA	English	1998	2020	884
Capital Research Center	USA	English	2000	2020	3665
Competitive Enterprise Institute	USA	English	1998	2020	6261
Foundation for Research on Economics & the Environment	USA	English	1998	2020	834
National Center for Public Policy Research	USA	English	1998	2020	7869
Reason Foundation	USA	English	1998	2020	423
Science and Public Policy Institute	USA	English	1999	2019	828
American Council on Science and Health	USA	English	1998	2020	12143
American Enterprise Institute	USA	English	1998	2020	3061
CATO Institute	USA	English	1998	2020	37268
CFACT	USA	English	2000	2020	4912
Frontiers of Freedom	USA	English	2008	2020	3046
Fraser Institute	Canada	English	1998	2020	5014
FreedomWorks	USA	English	1998	2020	3771
Heartland Institute	USA	English	1998	2020	18787
Heritage Foundation	USA	English	1998	2020	873
Hudson Institute	USA	English	2004	2020	152
Manhattan Institute	USA	English	1998	2020	1049
Pacific Research Institute for Public Policy	USA	English	2009	2020	485
Washington Policy Center	USA	English	2007	2020	4212

Table S5: Corpus of climate change-related blog posts (N = 139,912)

Blog Name	Country	Language	Year min	Year max	Docs
bobtisdale.wordpress.com	USA	English	2016	2020	15
c3headlines.com	USA	English	2008	2020	2436
carbon-sense.com	Australia	English	1999	2020	754
chiefio.wordpress.com	USA	English	2009	2020	3253
climate-resistance.org	UK	English	2002	2016	557
climate-skeptic.com	USA	English	2007	2016	628
climateaudit.org	Canada	English	2000	2020	2842
climatechangedispatch.com	Iceland	English	2003	2020	9642
climateconversation.org.nz	New Zealand	English	2005	2020	1252
climatesanity.wordpress.com	USA	English	2007	2016	197
climatescienceinternational.org	New Zealand	English	2010	2019	34
co2science.org	USA	English	1998	2020	6132
drroyspencer.com	USA	English	2008	2020	959
galileomovement.com.au	Australia	English	2011	2012	26
hockeyschtick.blogspot.com	USA	English	2009	2018	2875
joannenova.com.au	Australia	English	2000	2020	3856
judithcurry.com	USA	English	2010	2020	1996
junkscience.com	USA	English	1998	2020	10272
manicbeancounter.com	USA	English	2008	2020	569
masterresource.org	USA	English	2008	2020	2837
motls.blogspot.com	Czech Republic	English	2004	2020	7834
noconsensus.wordpress.com	USA	English	2008	2020	1427
nofrakkingconsensus.com	USA	English	2009	2020	1130
notalotofpeopleknowthat.wordpress.com	USA	English	2011	2020	6100
notrickszone.com	Germany	English	2010	2020	4148
principia-scientific.org	Iceland	English	2010	2020	6443
rationaloptimist.com	USA	English	2010	2020	719
realclimatescience.com	USA	English	2015	2020	6821
stevengoddard.wordpress.com	USA	English	2010	2016	23513
tallbloke.wordpress.com	USA	English	2009	2020	5146
thelukewarmersway.wordpress.com	USA	English	2012	2019	513
warwickhughes.com	Australia	English	2005	2020	1691
wattsupwiththat.com	USA	English	2006	2020	23295

Table S6: Linear regression results of donations from "key" donors and claim prevalence (as visualized in Fig. 4)

	Dependent variable:		
	Claim 5 Share	Claim 4 Share	Claims 1-3 Share
	(1)	(2)	(3)
Share of total revenue from key donors	0.403***	−0.608***	0.205**
	(0.104)	(0.156)	(0.088)
Constant	−0.017	0.989***	0.027
	(0.067)	(0.101)	(0.057)
Observations	14	14	14
R^2	0.555	0.557	0.311
Adjusted R^2	0.518	0.521	0.254
Residual Std. Error (df = 12)	0.091	0.137	0.077
F Statistic (df = 1; 12)	14.964***	15.115***	5.422**
Note:			*p<0.1; **p<0.05; ***p<0.01
